# Predicting Long-Term Benefits of Micro-Fragmented Adipose Tissue Therapy in Knee Osteoarthritis: Three-Year Follow-Up on Pain Relief and Mobility

**DOI:** 10.3390/jcm14134549

**Published:** 2025-06-26

**Authors:** Nicolae Stanciu, Nima Heidari, Mark Slevin, Alexandru-Andrei Ujlaki-Nagi, Cristian Trâmbițaș, Emil-Marian Arbănași, Octav Marius Russu, Răzvan Marian Melinte, Leonard Azamfirei, Klara Brînzaniuc

**Affiliations:** 1 Doctoral School of Medicine and Pharmacy, George Emil Palade University of Medicine, Pharmacy, Science and Technology of Targu Mures, 540139 Targu Mures, Romania; nicolae.stanciu@umfst.ro (N.S.); emil.arbanasi@umfst.ro (E.-M.A.); 2 Department of Anatomy, George Emil Palade University of Medicine, Pharmacy, Science and Technology of Targu Mures, 540139 Targu Mures, Romania; klara.brinzaniuc@umfst.ro; 3 Medical Supercomputation and Machine Learning, European Quantum Medical, London E10 5NP, UK; n.heidari@gmail.com; 4 Foot, Ankle and Limb Reconstruction, London W1G 7ET, UK; 5 Regenerative Medicine Laboratory, Centre for Advanced Medical and Pharmaceutical Research (CCAMF), George Emil Palade University of Medicine, Pharmacy, Science and Technology of Targu Mures, 540139 Targu Mures, Romania; 6 Psychiatry Clinic 2, Mures County Clinical Hospital, 540139 Targu Mures, Romania; u.alex8@yahoo.com; 7 Department of Plastic Surgery, George Emil Palade University of Medicine, Pharmacy, Science and Technology of Targu Mures, 540139 Targu Mures, Romania; cristian.trambitas@umfst.ro; 8 Department of Vascular Surgery, George Emil Palade University of Medicine, Pharmacy, Science and Technology of Targu Mures, 540139 Targu Mures, Romania; 9 Clinic of Vascular Surgery, Mures County Emergency Hospital, 540136 Targu Mures, Romania; 10 Department of Orthopedics and Traumatology I, George Emil Palade University of Medicine, Pharmacy, Science and Technology of Targu Mures, 540139 Targu Mures, Romania; octav.russu@umfst.ro; 11 Department of Orthopedics and Traumatology, Clinical County Hospital of Mureș, 540139 Targu Mures, Romania; 12 1st Department of Orthopedics and Traumatology, “Iuliu Hațieganu” University of Medicine and Pharmacy, 8 Victor Babes Street, 400012 Cluj-Napoca, Romania; rmmelinte@yahoo.de; 13 Department of Anesthesiology and Intensive Care Medicine, George Emil Palade University of Medicine, Pharmacy, Science and Technology of Targu Mures, 540139 Targu Mures, Romania; leonard.azamfirei@umfst.ro

**Keywords:** micro-fragmented adipose tissue, knee osteoarthritis, predictive value, orthobiologic treatment

## Abstract

**Objectives:** This study aims to assess the clinical efficacy of micro-fragmented adipose tissue (MFAT) therapy over three years in patients with KOA and to determine whether short-term improvements at three months can forecast long-term outcomes. **Methods:** A retrospective, observational study was conducted on 335 patients diagnosed with KOA who received a single MFAT injection. The patients were followed up at 3 months, 6 months, 1 year, 2 years, and 3 years, with assessments using the Visual Analog Scale (VAS), Oxford Knee Score (OKS), Western Ontario and McMaster Universities Osteoarthritis Index (WOMAC), and Knee Injury and Osteoarthritis Outcome Score (KOOS). Statistical analysis was performed to assess the differences in preoperative and postoperative scores (VAS, OKS, WOMAC, KOOS) to evaluate the predictive role of 3-month score changes on long-term clinical outcomes. **Results:** All measured scores (VAS, OKS, WOMAC, KOOS) showed significant improvement at 3 months, with sustained improvements through 3 years (*p* < 0.001). Early score changes at 3 months were significantly associated with improved clinical outcomes at 1, 2, and 3 years (*p* < 0.05). Logistic regression confirmed early post-treatment improvements as independent predictors of long-term benefit, except for the VAS score at 3 years (*p* = 0.098). A comparative analysis between completers and dropouts showed no baseline differences; however, significant outcome differences emerged at later follow-up points. Due to insufficient data at the 3-year mark among dropouts, statistical comparisons were not possible for that time point. **Conclusions:** MFAT treatment was associated with consistent symptomatic improvement in patients with KOA, and early clinical response at 3 months served as a reliable predictor of long-term pain and function outcomes. While this study focused on patient-reported symptom relief and not structural regeneration, the results support MFAT as a minimally invasive option for symptom management. Early post-treatment response may serve as a useful tool for clinicians to predict long-term therapeutic success and personalize treatment strategies for KOA patients.

## 1. Introduction

Osteoarthritis (OA) represents the main cause of joint degeneration, affecting over 200 million patients globally, with rising numbers anticipated due to population aging and lifestyle changes [1]. It is also one of the significant contributors to years lived with disability among musculoskeletal conditions [2]. A comprehensive knowledge of OA highlights its increasing clinical and social burden, attributed not only to its prevalence but also to the complex interaction of variables that accelerate joint degradation [3].

Knee osteoarthritis (KOA) and hip osteoarthritis (HOA) have the highest prevalence and are the leading causes of disability, affecting the functionality and, implicitly, the quality of life of patients [1,4,5]. KOA is a wide-ranging, degenerative joint condition marked by the gradual structural decline of the whole joint system, involving articular cartilage, subchondral bone, ligaments, synovium, and periarticular muscles [6]. It affects over 37% of people aged 60 and above, with a notably greater incidence in women, and it constitutes a significant public health challenge because of its correlation with chronic disability, limited physical activity, and decreased quality of life [7]. The pathophysiology of KOA is multifaceted, shaped by both internal and extrinsic risk factors. Significant parameters include advanced age, female gender, obesity, prior musculoskeletal injuries (e.g., anterior cruciate ligament rupture or meniscal tears), and occupational exposures characterized by repeated joint loading, such as squatting, kneeling, or heavy lifting. Obesity elevates mechanical stress on the knee joint and fosters systemic inflammation, hence intensifying disease development. Anatomical and hormonal variables may partially explain the observed sex differences in the occurrence and severity of KOA. These determinants, combined with the often discordant relationship between radiographic findings and clinical symptoms, highlight the heterogeneity of KOA and the necessity for individualized multimodal treatment strategies [6].

KOA can be managed with both non-surgical treatments (OARSI Guidelines), including patient education, structured land-based exercise, weight management, topical and oral NSAIDs (as appropriate), intra-articular injections [8,9,10], and surgical methods, each according to multiple variables, such as disease severity, patient characteristics, and risk profiles.

The surgical management of KOA encompasses a range of procedures including arthroscopic debridement, cartilage repair techniques, osteotomies, and knee arthroplasty, each selected based on severity, compartmental involvement, patient age, and comorbidities [11,12]. Particularly, total knee arthroplasty (TKA) remains a well-established option for patients with advanced disease who are unresponsive to conservative measures [13]. While these interventions can offer meaningful symptom relief and functional improvement, they are not without risks [14,15,16,17,18]. Some of the potential complications can vary from persistent postoperative pain, stiffness, instability, malalignment, extensor mechanism disruption, prosthesis loosening or failure, perioperative infection, neurovascular injury, acute renal failure, or thromboembolic events, some of which could be life-threatening [14,15,16,17,18]. These adverse events may be exacerbated by patient-specific factors such as obesity, advanced age, or poor bone quality. Furthermore, surgical candidacy must be carefully evaluated; absolute and relative contraindications, such as significant comorbidities, can limit the appropriateness of certain procedures. Consequently, surgical decision-making must be tailored, weighing the potential benefits against the individual patient’s risk profile and functional expectations. Therefore, apart from well-documented cases where surgical intervention was unequivocally required with minimal risks associated, it became imperative to investigate alternative therapeutic strategies. Consequently, research initiatives focused on creating and assessing non-surgical methods to alleviate symptoms and slow down disease progression in patients for whom surgical interventions presented a greater risk.

Studies in regenerative medicine have emphasized the potential of mesenchymal stem cells (MSCs) as a highly effective therapeutic approach for chondral degeneration [19,20,21]. Micro-fragmented adipose tissue (MFAT) is considered superior to other biological treatments in KOA due to its high concentration of MCSs derived from adipose tissue [22,23,24]. MFAT treatment involves a two-step protocol, involving adipose tissue harvesting via mini-liposuction, followed by intra-articular injection of the processed product. While both steps involve tissue access, the intervention is consistently described in the literature as minimally invasive due to its outpatient setting, use of local anesthesia, small incisions, and reduced recovery time [25,26,27]. Unlike MSCs from bone marrow, which are difficult to obtain in significant quantities and involve painful procedures, adipose tissue provides a much more substantial source of MSCs with minimal side effects [28,29]. These MSCs obtained from MFAT display substantial immunomodulatory, anti-inflammatory, and regenerative effects, which stimulate localized healing, making MFAT an efficient option for KOA management [30,31,32,33,34,35,36].

The Lipogems^®^ system (Lipogems International SpA, Milan, Italy) used to process adipose tissue was FDA 510(k) cleared for general orthopedic and arthroscopic applications, supporting its safe and standardized use in clinical settings [37,38]. MFAT has been widely utilized in orthopedic practice as a minimally invasive therapeutic option, particularly for the symptomatic management of knee osteoarthritis [39].

The aim of this study is to evaluate the clinical effectiveness of MFAT therapy over a three-year period in patients with KOA and to determine whether short-term improvements at three months can predict long-term outcomes.

## 2. Materials and Methods

### 2.1. Study Design

This study conducted a retrospective review of prospectively collected data to evaluate longitudinal symptomatic outcomes, specifically pain and functional scores, following a single injection of MFAT in patients diagnosed with KOA. The study was conducted in a private orthopedic clinic and included patients who presented with knee pain and had a confirmed diagnosis of knee osteoarthritis. All patients were reviewed by an orthopedic surgeon and were informed of all possible options for treating KOA. Prior to initiating treatment, all participants were thoroughly informed about the full range of therapeutic options available for managing their condition. These included conservative management strategies, injectable therapies such as corticosteroids, hyaluronic acid (HA), platelet-rich plasma (PRP), and MFAT, as well as surgical interventions including osteotomy, partial knee replacement, and total knee arthroplasty.

Patients provided informed consent to undergo the MFAT treatment and standardized assessments measuring pain, joint stiffness, physical function, and quality of life. They were not involved in the design, implementation, or planning of the study. As part of the consent process, patients authorized the designated physician to perform the indicated medical treatment or procedure as described in the consent documentation. The nature of the intervention, its potential benefits, and expected outcomes were clearly explained. Additionally, patients acknowledged and agreed that anonymized data from their clinical evaluations could be used for research and scientific publication purposes, with all identifying information removed to ensure confidentiality. The research was conducted following the principles of Good Clinical Practice (NIHR) and the General Medical Council (GMC) guidelines concerning research, patient consent, and future publications while also adhering to the Declaration of Helsinki. This study took place in a private practice environment. Approval was granted by the Research Ethics Committee of the George Emil Palade University of Medicine, Pharmacy, Science and Technology in Targu Mures, Romania (no. 3365/2024, 16 October 2024).

### 2.2. Inclusion Criteria

The patients included in the research were diagnosed with KOA; they visited a private clinic and received a single injection of MFAT. All patients underwent either weight-bearing anteroposterior (AP) and lateral (LL) knee X-rays or MRI, with long-leg standing radiographs performed in selected cases to assess lower limb alignment and confirm eligibility based on the exclusion criteria. Radiographic grading was conducted using the Kellgren–Lawrence (KL) classification system [40].

Patients with a deformity exceeding 10 degrees of varus or valgus, a recent injury (within the last 3 months) to the symptomatic knee, infectious joint disease, alignment issues, pregnancy, anticoagulation therapy, thrombocytopenia, coagulation disorders, intra-articular steroid injections conducted within the past three months, and HA injections received before this treatment were excluded.

### 2.3. Data Collection

A total number of 335 patients were enrolled, with an average age of 64.67 years; among them, 186 were men (55.52%) and 149 were women (44.48%). Pre-operative and post-operative evaluations were conducted during scheduled visits at 3 months, 6 months, 1 year, 2 years, and 3 years. At each evaluation, the following assessments were performed: the Visual Analog Score (VAS) [41,42] to evaluate the severity of pain; the Oxford Knee Score (OKS) [43] for a functional assessment; the Western Ontario and McMaster Universities Osteoarthritis Index (WOMAC) [42,44] for pain, stiffness, and physical function; and the Knee Injury and Osteoarthritis Outcome Score (KOOS) for pain, symptoms, functionality in daily living, engagement in sports and recreational activities, and knee-related quality of life [44].

Demographic data for each patient, including age and sex, as well as body mass index (BMI), the severity of KOA according to the Kellgren–Lawrence classification [26], and the location of the affected knee, were obtained from the electronic database of the private clinic. Moreover, the pre-operative and postoperative (at intervals of 3 months, 6 months, 1 year, 2 years, and 3 years) VAS, OKS, WOMAC, and KOOS score values were obtained from the electronic database of the private clinic (Table 1).

### 2.4. MFAT Technique

Comprehensive and informed consent was obtained for each aspect of the procedure, including sedation, liposuction, and image-guided intra-articular injection. All procedures were conducted in an operating theater on a day-case basis. Adipose tissue was harvested and microfragmented using a previously published technique [33,36,45,46].

This injection was performed as an outpatient procedure under local anesthesia. A 17G blunt cannula attached to a Luer-lock 60 cc syringe was inserted through a small incision into the subcutaneous fat of the lower abdomen to extract adipose tissue, allowing for the injection of 50 mL aliquots of 150–200 mL of Klein sterile solution (lignocaine, adrenaline, and 0.9% sodium chloride). A 13G blunt cannula attached to a Vaclock 20 mL syringe was used to manually extract approximately 50 mL of adipose tissue. The lipoaspirate was processed using a single-use device (Lipogems^®^ system) that mechanically fragmented the adipose tissue, removed contaminants (such as blood and debris), and filtered the product within 20 min. The resulting solution comprised approximately 33% pericytes, corresponding to roughly 9 × 10^3^ cells per ml of MFAT, with an estimated ratio of 1:100 cells from raw adipose tissue identified as stem cells compared to a ratio of approximately 1:100,000 when sourced from bone marrow. Under ultrasound guidance, 6–8 mL of the resulting MFAT was then injected directly into the suprapatellar pouch of the knee joint, immediately proximal to the patella.

### 2.5. Statistical Analysis

Statistical analysis was conducted using SPSS for Mac OS version 28.0.1.0 (SPSS, Inc., Chicago, IL, USA). Age, BMI, VAS, OKS, KOOS, and WOMAC score values are reported as mean values with standard deviation (SD). To assess the differences in continuous variables and changes between preoperative and postoperative scores throughout the follow-up period, we applied the Mann–Whitney test and Student’s *t*-test. The changes in the scores at 3 months (∆VAS Pain Score, ∆OKS, ∆WOMAC Score, ∆KOOS Pain Score, ∆KOOS Symptom Score, ∆KOOS Daily Activities Score, ∆KOOS Sport Score, ∆KOOS) were calculated as the difference between the values at 3 months postoperatively and the preoperative values for OKS and KOOS, respectively, and the difference between the preoperative values and those obtained 3 months postoperatively for VAS and WOMAC scores. Furthermore, clinical improvement at one year, two years, and three years was defined as a positive difference between the scores recorded during the aforementioned visits and the preoperative scores. Additionally, we employed receiver operating characteristic (ROC) curve analysis to investigate the association between the changes in VAS, OKS, KOOS, and WOMAC scores with clinical improvement at one year, two years, and three years. We performed univariate regression analyses to evaluate the predictive role of short-term (3-month) changes in the recorded scores and the mid- and long-term clinical benefits after treatment with MFAT for KOA patients. All tests were two-tailed, and a *p*-value less than 0.05 was considered statistically significant.

## 3. Results

In the current study, we enrolled 335 patients with an average age of 64.67 ± 1.40, the majority of whom were male (55.52%) (Table 1). The patients had an average BMI of 27.57 ± 4.64, with 51.94% of them having KOA lesions at the level of the right knee and 48.06% at the level of the left knee. Regarding clinical stage, 4.78% of patients had KL Grade I, 20.30% had KL Grade II, 24.78% had KL Grade III, and 48.36% had KL Grade IV (Table 2).

**Figure 1:**
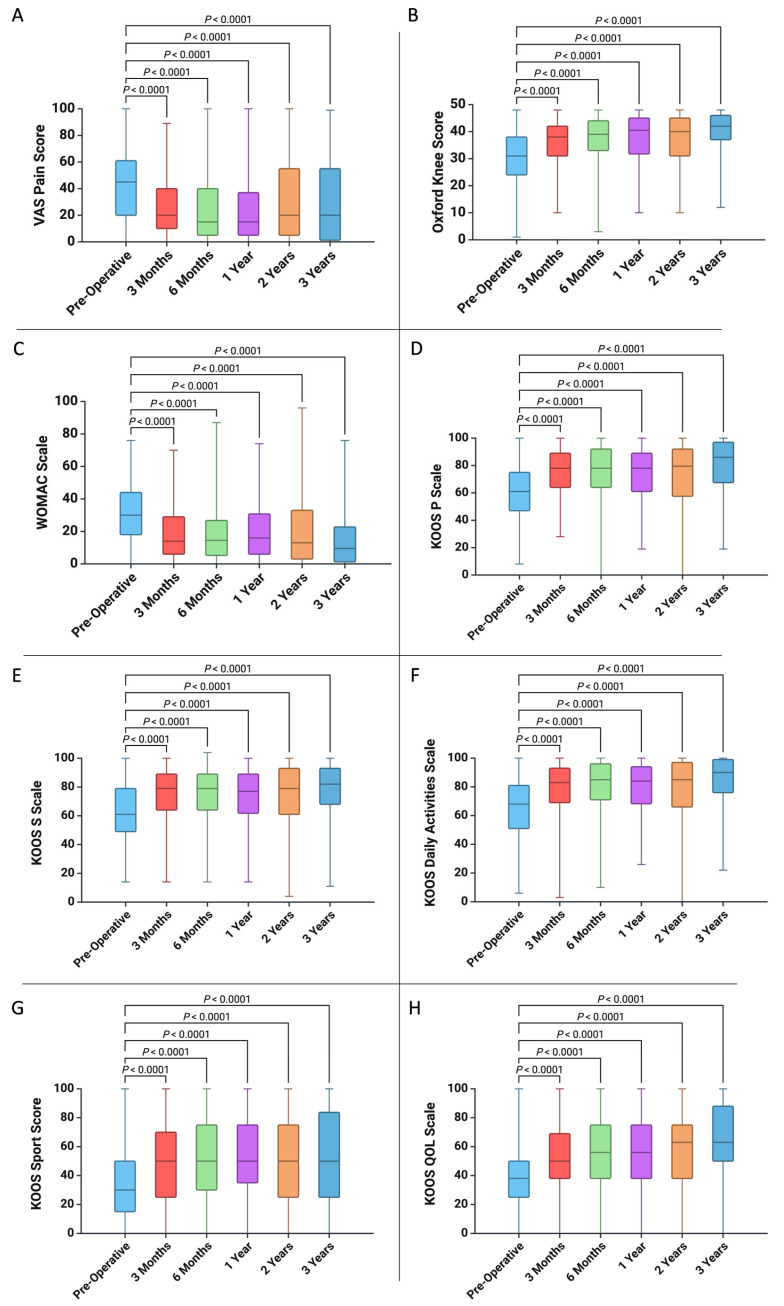
The benefit of MFAT treatment up to 3 years post-treatment quantified by (**A**) VAS Pain Score, (**B**) Oxford Knee Score, (**C**) WOMAC Scale, (**D**) KOOS P Scale, (**E**) KOOS S Scale, (**F**) KOOS Daily Activities Scale, (**G**) KOOS Sport Score, and (**H**) KOOS QOL Scale.

Furthermore, we analyzed the improvement in quality of life, pain, and functionality by measuring the VAS Pain Score, OKS, WOMAC Score, KOOS Pain Score, KOOS Symptom Score, KOOS Daily Activities Score, KOOS Sport Score, and KOOS QOL Score before the MFAT treatment and at 3 months, 6 months, 1 year, 2 years, and 3 years post-operation (Figure 1 and Table 3). As shown in Figure 1, we observed improvements in all the analyzed scores up to 3 years compared to pre-surgery status (for all *p* < 0.001).

During the three-year follow-up, we identified a significant number of patient dropouts. To prevent overestimating the treatment effect, we compared the baseline characteristics of patients based on their attendance at the three-year follow-up visit (Supplementary Materials Table S1). Notably, there were no significant differences in age or sex between the complete group and the dropouts, except for a higher proportion of males in the VAS Pain Score dropout group (*p* = 0.002). Similarly, no differences were observed between the groups for any of the scores related to BMI or the affected knee, and we did not find any differences related to clinical severity. Regarding the pre-operative value, no differences were observed concerning the VAS Pain Score, OKS, KOOS Pain Score, KOOS Symptom Score, and KOOS QOL Score. However, the dropout patients exhibited a higher pre-operative WOMAC Score at baseline (*p* = 0.037) and a lower KOOS Daily Activities Score (*p* = 0.045) and KOOS Sport Score (*p* = 0.039) at baseline (Supplementary Materials Table S1).

**Figure 2:**
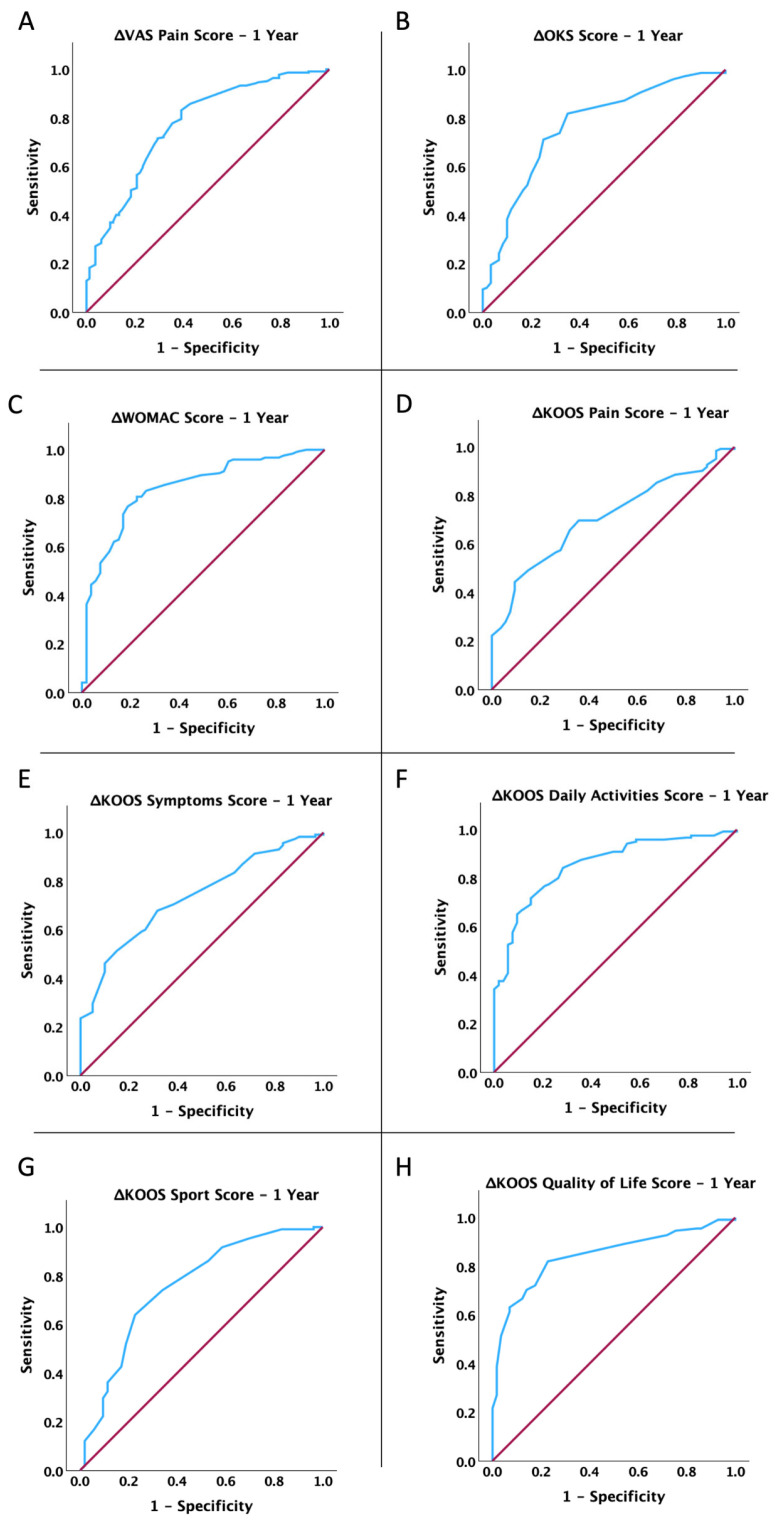
ROC curve analysis: association between changes in dynamics (3 months) and clinical improvement 1-year post-treatment with MFAT for (**A**) VAS Pain Score, (**B**) Oxford Knee Score, (**C**) WOMAC Score, (**D**) KOOS P Score, (**E**) KOOS S Score, (**F**) KOOS Daily Activities Score, (**G**) KOOS Sport Score, and (**H**) KOOS QOL Score.

**Figure 3:**
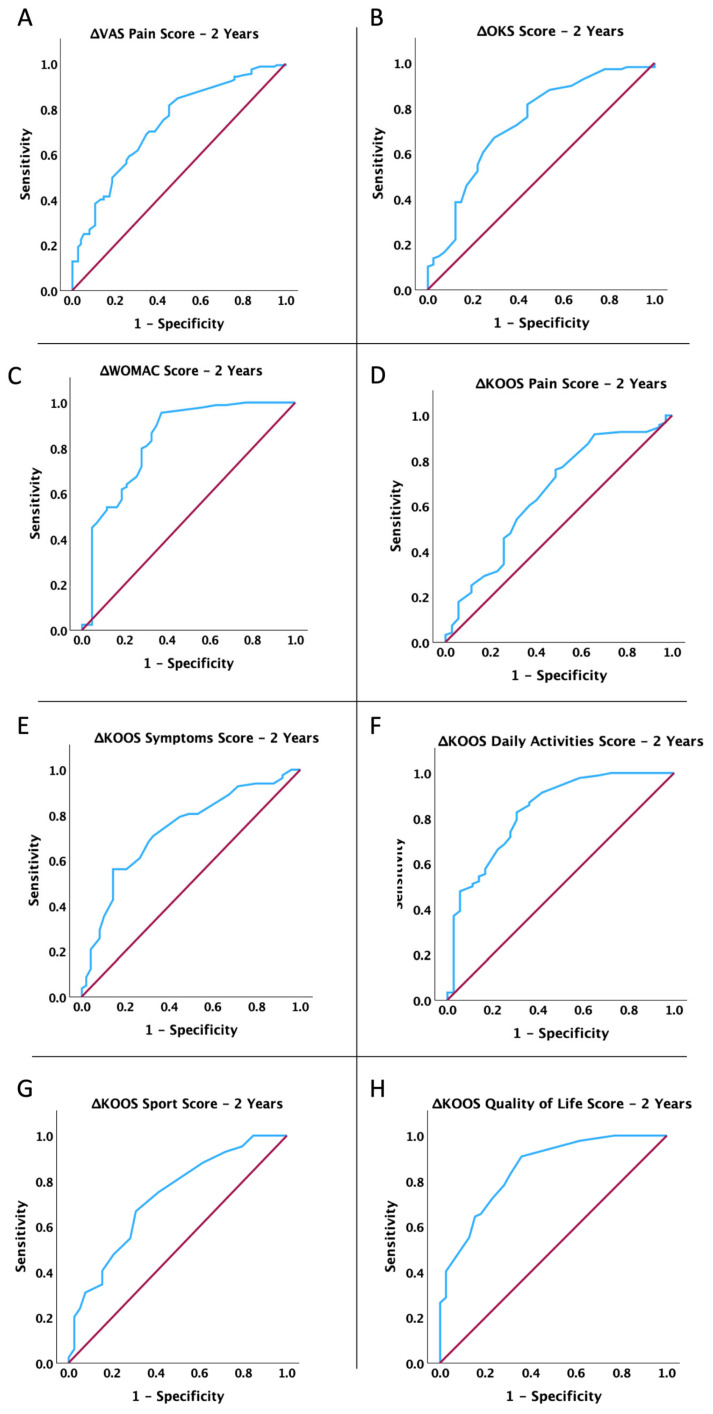
ROC curve analysis: association between changes in dynamics (3 months) and clinical improvement 2 years post-treatment with MFAT for (**A**) VAS Pain Score, (**B**) Oxford Knee Score, (**C**) WOMAC Score, (**D**) KOOS P Score, (**E**) KOOS S Score, (**F**) KOOS Daily Activities Score, (**G**) KOOS Sport Score, and (**H**) KOOS QOL Score.

**Figure 4:**
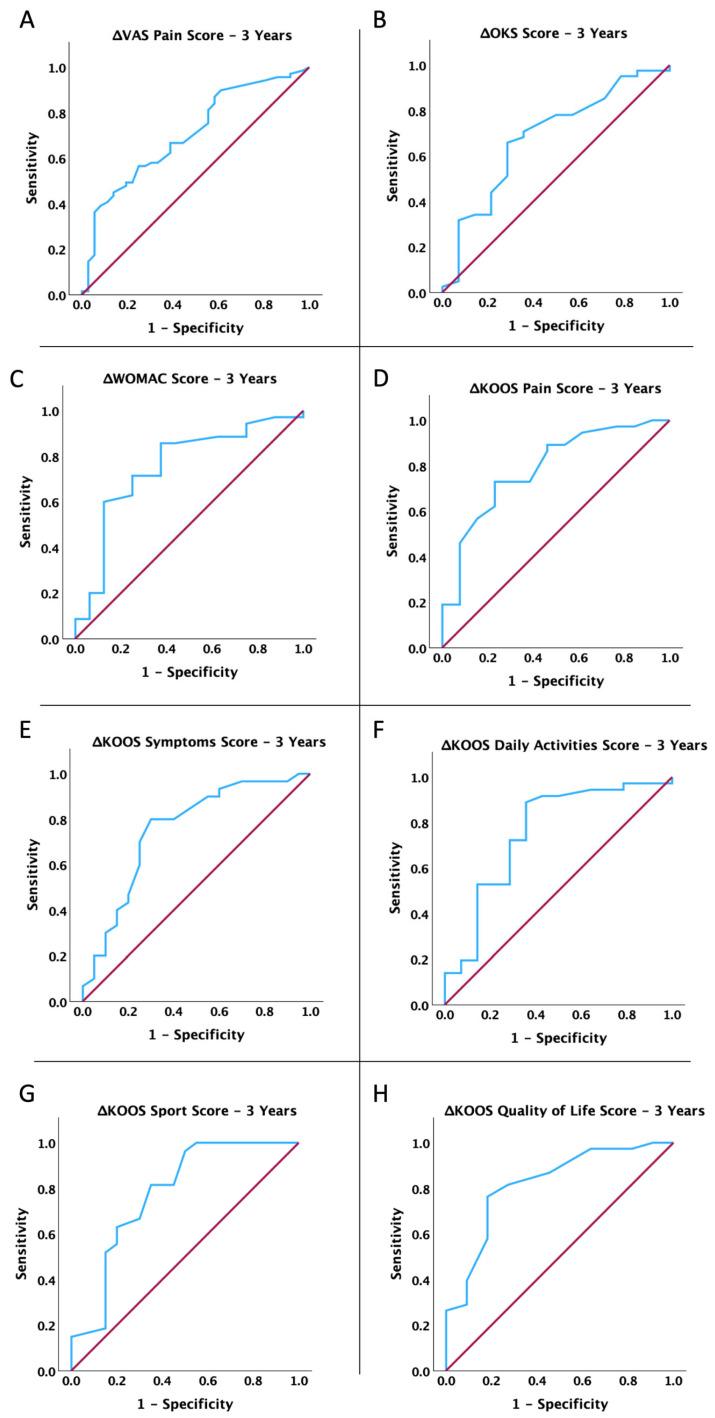
ROC curve analysis: association between changes in dynamics (3 months) and clinical improvement 3 years post-treatment with MFAT for (**A**) VAS Pain Score, (**B**) Oxford Knee Score, (**C**) WOMAC Score, (**D**) KOOS P Score, (**E**) KOOS S Score, (**F**) KOOS Daily Activities Score, (**G**) KOOS Sport Score, and (**H**) KOOS QOL Score.

Moreover, we conducted an ROC analysis to determine if the short-term (3 months) improvement in mobility, pain, and functionality of the affected limb after MFAT treatment was linked to lasting benefits at 1, 2, and 3 years. We calculated the dynamic change in score at 3 months as the differences between the pre-operative score and the score at 3 months for the VAS Pain Score and WOMAC Score and as differences between the 3-month and pre-operative scores for the other determined scores. As shown in Figure 2, Figure 3 and Figure 4 and Table 4, the dynamic change in all scores at 3 months post-operation is significantly associated with the benefits of MFAT treatment at 1 year, 2 years, and 3 years (for all *p* < 0.05).

To analyze the predictive role of changes in the scores at 3 months after treatment compared to the pre-operative stage and their correlation with clinical improvement at 1 year, 2 years, and 3 years, we conducted binary logistic regression. The results in Table 5 show that the clinical improvement of patients at 3 months after treatment with MFAT is a strong predictor of clinical benefit at 1 year (*p* < 0.001), 2 years (*p* < 0.05), and 3 years (*p* < 0.05), except for the ∆VAS Pain Score at 3 years (*p* = 0.098).

## 4. Discussion

The primary outcome of this study was the demonstrated association between early clinical improvement and sustained therapeutic benefit in patients with KOA following MFAT treatment. Our analysis revealed that improvements observed at 3 months post-treatment were strongly predictive of favorable clinical outcomes at 1, 2, and 3 years. Specifically, improvements in pain and functional scores at this early time point served as robust indicators of long-term therapeutic efficacy. A comparative analysis between patients who completed the 3-year follow-up and those who dropped out earlier revealed no significant differences in baseline clinical scores (VAS, OKS, KOOS, WOMAC), confirming group comparability at study entry. Furthermore, the effectiveness of MFAT treatment persisted over the three-year follow-up period, with all outcome measures showing significant improvement relative to the baseline, regardless of the specific scoring system used.

Subgroup analyses by age, sex, BMI, and radiographic stage were not conducted within the scope of the present study; these factors have been comprehensively explored in previous publications involving the cohort. These studies employed multivariate models and bias-mitigated algorithms to evaluate demographic and clinical predictors of responses to MFAT, thereby supporting the relevance of these variables in personalized treatment. Gender-specific outcomes revealed that female patients experienced significantly higher improvements in pain and function compared to male patients. Specifically, after the 2-year follow-up, the mean OKS improvement in females was +12.2 (95% CI: +10.3 to +14.1) compared to +4.6 (95% CI: +2.5 to +6.8) in males. Similarly, the mean reduction in pain as evaluated by the VAS score was −28.8 (95% CI: −34.4 to −23.6) in females versus −9.7 (95% CI: −16.0 to −3.0) in males. Age-stratified analysis demonstrated functional improvement in all groups, with males under 65 years (from 30.5 to 38.4) and females 65–75 years (from 25.0 to 37.8) showing the greatest mean OKS increases. Functional gains were still observed in individuals over 75 years old (females: 24.9 to 31.5; males: 30.2 to 32.2), demonstrating efficacy even in this age range. When stratified by disease severity, both KL grade 3 and grade 4 patients demonstrated improved function. However, the pattern of change differed. OKS decreased at 12 and 24 months in females with KL grade 3 and males with KL grade 4, indicating that long-term responses varied by illness stage. A BMI-based subgroup analysis revealed consistent improvements across categories, with no significant moderating effect of BMI on treatment outcomes [33,36,46]. These data revealed that MFAT’s therapeutic effects were modulated by gender, age, and radiographic severity, emphasizing the need to include multivariate factors in outcome analysis for biologic therapies in KOA. In this study, we focused specifically on the predictive value of early response while recognizing the importance of demographic moderators already established in prior work.

To comprehensively assess patient-reported outcomes, we employed multiple validated instruments, including VAS, OKS, WOMAC, and KOOS. Each of these measures demonstrated statistically significant improvements post-intervention (*p* < 0.001), underscoring MFAT’s multidimensional impact on pain, symptomatology, and functional capacity. The observed improvement demonstrated MFAT’s potential to positively influence pain relief, quality of life, and functional outcomes over a 3-year span. Notably, each score improved significantly compared to the pre-surgical status (*p* < 0.001), underscoring MFAT’s comprehensive effect on pain and functionality.

These findings align with the prior literature. For instance, Van Genechten et al. [47] reported early improvements in pain and function following MFAT injections, reinforcing the short-term efficacy of adipose-derived therapies in KOA management. Similarly, Onorato et al. [48] demonstrated durable clinical improvement in 68% of patients at four years post-MFAT, underscoring the long-term viability of this intervention.

Ulivi et al. [49], in a randomized controlled trial, showed that combining MFAT with arthroscopic debridement led to substantial functional enhancements at 12 months, whereas our findings suggested that MFAT alone was sufficient to confer durable benefits.

The long-term safety and effectiveness of cell-based therapies have also been corroborated in studies such as that by Kim et al. [50], who observed sustained pain and functional improvement five years after intra-articular MSC administration. Guo et al. [51] identified novel anti-inflammatory and angiogenic properties of MFAT, which may have contributed to its therapeutic effects in clinical settings.

In a study by Heidari et al., MFAT was proposed as an alternative to total knee replacement (TKR) in patients with advanced KOA, demonstrating significant improvement at two years. Their findings supported the role of MFAT in potentially delaying the need for surgical intervention [33].

Importantly, our findings provided empirical support for the predictive framework proposed by the precisionKNEE system, a gender bias-mitigated data-driven tool designed to optimize patient selection for biological treatments in advanced KOA [35]. The precisionKNEE model incorporated demographic variables, disease severity, and early treatment response metrics to predict long-term outcomes. Our data validated this approach by showing that standard patient-reported outcome measures collected at 3 months (e.g., VAS, WOMAC, KOOS) were significantly correlated with outcomes at 1, 2, and 3 years. This underscored the clinical value of early symptom tracking in informing patient-specific therapeutic strategies.

While the precisionKNEE platform primarily emphasized pre-treatment decision-making, our study extended its applicability by demonstrating that the longitudinal monitoring of post-treatment outcomes can further enhance predictive modeling. The clear correlation between short-term clinical gains and sustained improvement suggests that integrating early response metrics into predictive algorithms may refine patient stratification and optimize treatment regimens. Such a framework holds broader relevance for regenerative medicine, where the early identification of responders can guide real-time therapeutic adjustments and improve overall care quality. Future research should aim to integrate real-time patient-reported outcomes with machine learning and AI-based analytics to advance precision medicine in KOA management [35].

Although clinical outcome measures, including pain and functional scores, offered valuable insights into patient-perceived benefits, they did not provide direct evidence of cartilage tissue regeneration. Advanced imaging modalities and novel diagnostic techniques were considered to offer more accurate and objective evaluations in this context. Magnetic resonance imaging (MRI) was regarded as the benchmark for a non-invasive assessment of cartilage morphology, facilitating the identification of softening, fibrillation, and structural abnormalities, even in initial stages. High-resolution 3.0T MRI and sophisticated grading systems based on the International Cartilage Repair Society criteria facilitated the classification of cartilage damage and the assessment of reparative tissue following biological therapies. Nonetheless, MRI had limitations, such as elevated costs, reliance on operator skill, and inconsistent sensitivity during the early stages of disease [52].

On the other hand, vibroarthrography (VAG) offered a dynamic, cost-effective, and non-invasive approach for evaluating the mechanical integrity of articular cartilage. VAG indicated surface roughness and lubrication status by measuring the acoustic signals produced during joint movement, which were closely associated with early cartilage degeneration. Recent research suggested that VAG, particularly when integrated with signal processing algorithms and machine learning models, could achieve elevated diagnostic precision in differentiating healthy cartilage from degenerated cartilage [53].

Particularly, the integration of advanced signal processing techniques, such as ensemble empirical mode decomposition (EEMD) and detrended fluctuation analysis (DFA), has allowed for the extraction of detailed time–frequency features from acoustic signals generated during joint movement. When these features were further analyzed using convolutional neural networks (CNNs), the diagnostic accuracy for differentiating healthy from degenerated cartilage reached levels as high as 99% [54]. Utilizing VAG as a screening or follow-up instrument in conjunction with imaging and clinical evaluations potentially improved early detection, monitored tissue response to therapy, and augmented the assessment of regenerative outcomes [54,55,56]. To achieve a more comprehensive assessment of MFAT’s therapeutic potential, future studies should integrate these diagnostic approaches with biochemical and biomechanical markers. Moreover, the inclusion of control groups and extended follow-up periods will be essential to further clarify MFAT’s role in KOA management and to substantiate its regenerative effects through objective mechanistic validation.

### Limitations

Despite its strengths, the study had several limitations. The research did not seek to investigate the regenerative or structural effects of MFAT; therefore, no claims were made regarding cartilage repair or tissue regeneration. The analysis was exclusively centered on evaluating subjective symptom relief, assessed using validated outcome measures such as the VAS, KOOS, OKS, and WOMAC scores and on determining the prognostic value of short-term improvements for sustained therapeutic benefit.

This study did not apply standardized responder definitions such as the OMERACT-OARSI criteria or minimal clinically important improvement (MCII) thresholds. Due to its retrospective nature, the analysis focused on reporting absolute changes in validated outcome scores (VAS, OKS, KOOS, WOMAC) across follow-up intervals. While this limits direct comparison with therapeutic response rates (TRR%) reported in prospective trials, we note that MCII thresholds and response stratification have been applied in previous publications derived from this cohort [33,36,46], where multivariate analyses were used to identify demographic and clinical predictors of meaningful improvement. These findings support the clinical relevance of the outcome measures used, even in the absence of formal responder classification in the present analysis.

A key limitation of this study is the absence of a control group, which precludes the definitive attribution of clinical improvements to MFAT therapy alone. The lack of comparison with a placebo, standard care, or other biological treatments limits the ability to account for potential confounders or placebo effects. Future research should address this gap through prospective, randomized controlled trials to validate these findings and establish comparative efficacy.

## 5. Conclusions

In managing KOA, treatment options range from conservative treatment to surgical interventions. However, surgical procedures carry significant risks, including infection, neurovascular injury, loss of biomechanical stability, and life-threatening complications, alongside absolute or relative contraindications. Consequently, there is a pressing need to identify effective, lower-risk alternatives for patients who present a high risk of complications, aiming to alleviate symptoms and attenuate the progression of the disease.

This study provided compelling evidence that early clinical improvement was a key predictor of long-term benefits throughout all 3 years. These findings support the use of early post-treatment response as a clinically meaningful indicator of long-term therapeutic success. Furthermore, the consistent and statistically significant improvements across multiple validated clinical scores position MFAT as a promising and durable non-surgical treatment option for KOA.

These results underscore the clinical utility of early symptom tracking as a prognostic tool and highlight its potential to inform personalized patient-specific therapeutic strategies. By identifying early responders, clinicians may be better positioned to tailor interventions, optimize treatment pathways, and enhance long-term outcomes in KOA management.

## Figures and Tables

**Table 1:** The number of patients for whom the VAS, OKS, KOOS, and WOMAC score values pre-operative and during the follow-up period are available in the electronic database.

	Pre-Operative	3 Months	6 Months	1 Year	2 Years	3 Years
VAS	335	326	310	310	239	106
OKS	323	269	241	232	179	59
KOOS	312	250	214	202	160	54
WOMAC	314	252	214	202	160	54

**Table 2:** The data of the patients enrolled in this study.

Variables	All Patients (*n* = 335)
Age, mean ± SD	64.67 ± 11.40
Male, no. (%)	186 (55.52%)
Body mass index, mean ± SD	27.57 ± 4.64
Right knee, no. (%)	174 (51.94%)
Left knee, no. (%)	161 (48.06%)
Kellgren–Lawrence Classification	
Not available, no. (%)	6 (1.78%)
Grade I, no. (%)	16 (4.78%)
Grade II, no. (%)	68 (20.30%)
Grade III, no. (%)	83 (24.78%)
Grade IV, no. (%)	162 (48.36%)

**Table 3:** Functionality and pain scores recorded in the current study preoperatively and at 3 months, 6 months, 1 year, 2 years, and 3 years following the MFAT treatment.

Follow-Up	Pre-Operative		3 Months		6 Months		1 Year		2 Years		3 Years	
Score	Mean	SD	Mean	SD	Mean	SD	Mean	SD	Mean	SD	Mean	SD
VAS Pain Score	43.49	26.27	25.86	22.59	24.55	23.86	23.52	24.71	29.27	28.31	28.99	29.32
OKS	30.38	8.91	36.25	8.41	37.07	8.55	37.52	8.72	36.89	9.73	40.30	7.81
WOMAC Score	31.34	17.54	18.56	15.47	18.78	17.36	19.75	16.79	20.88	20.77	14.29	15.68
KOOS Pain Score	60.67	18.86	74.62	17.57	74.68	20.21	73.97	19.53	73.58	22.34	80.53	19.55
KOOS Symptom Score	62.56	19.02	74.89	17.89	74.82	19.32	74.52	18.89	74.41	21.83	79.21	18.56
KOOS Daily Activities Score	66.42	19.27	79.63	17.29	80.61	18.37	79.74	17.78	78.37	22.29	85.83	16.21
KOOS Sport Score	34.91	25.12	47.25	27.68	52.38	28.65	52.40	26.74	50.56	30.39	53.79	30.97
KOOS QOL Score	37.01	20.09	51.16	23.22	56.58	25.36	54.68	25.41	56.31	27.67	65.96	25.48

**Table 4:** The characteristics of the ROC curve analysis regarding the association of the short-time change (3 months) in the dynamics of the scores: VAS Pain Score, OKS, WOMAC Score, KOOS Pain Score, KOOS Symptom Score, KOOS Daily Activities Score, KOOS Sport Score, and KOOS QOL Score and clinical improvement in the medium and long term.

Variables	AUC	Std. Error	95% CI	*p* Value
1 Year Clinical Improvement				
∆VAS Pain Score	0.772	0.031	0.712–0.832	<0.001
∆OKS	0.766	0.037	0.694–0.838	<0.001
∆WOMAC Score	0.841	0.032	0.778–0.904	<0.001
∆KOOS Pain Score	0.708	0.039	0.631–0.785	<0.001
∆KOOS Symptom Score	0.734	0.038	0.661–0.808	<0.001
∆KOOS Daily Activities Score	0.855	0.030	0.797–0.913	<0.001
∆KOOS Sport Score	0.754	0.042	0.671–0.837	<0.001
∆KOOS QOL Score	0.844	0.030	0.785–0.904	<0.001
2 Years Clinical Improvement				
∆VAS Pain Score	0.730	0.035	0.662–0.799	<0.001
∆OKS	0.734	0.048	0.640–0.827	<0.001
∆WOMAC Score	0.834	0.041	0.752–0.915	<0.001
∆KOOS Pain Score	0.656	0.056	0.546–0.766	0.005
∆KOOS Symptom Score	0.734	0.045	0.646–0.822	<0.001
∆KOOS Daily Activities Score	0.831	0.042	0.749–0.914	<0.001
∆KOOS Sport Score	0.725	0.049	0.629–0.822	<0.001
∆KOOS QOL Score	0.852	0.036	0.780–0.923	<0.001
3 Years Clinical Improvement				
∆VAS Pain Score	0.704	0.053	0.600–0.807	<0.001
∆OKS	0.683	0.031	0.517–0.849	0.031
∆WOMAC Score	0.753	0.077	0.601–0.904	0.001
∆KOOS Pain Score	0.788	0.074	0.643–0.933	<0.001
∆KOOS Symptom Score	0.755	0.074	0.611–0.899	0.001
∆KOOS Daily Activities Score	0.757	0.084	0.592–0.921	0.002
∆KOOS Sport Score	0.779	0.073	0.636–0.922	<0.001
∆KOOS QOL Score	0.819	0.075	0.672–0.967	<0.001

**Table 5:** The association between the changes in the short-term dynamics (3 months) of the recorded scores and the clinical benefit in the medium and long term after treatment with MFAT for KOA.

Follow-Up	1-Year Clinical Improvement			2-Year Clinical Improvement			3-Year Clinical Improvement		
Score	OR	95% CI	*p* Value	OR	95% CI	*p* Value	OR	95% CI	*p* Value
∆VAS Pain Score	3.07	1.94–4.85	<0.001	2.44	1.51–3.94	<0.001	1.83	0.89–3.76	0.098
∆OKS	2.34	1.51–3.63	<0.001	1.81	1.15–2.86	0.011	4.42	1.48–13.14	0.008
∆WOMAC Score	2.77	1.75–4.39	<0.001	2.68	1.63–4.38	<0.001	2.73	1.25–6.01	0.012
∆KOOS Pain Score	5.96	3.10–11.47	<0.001	5.85	2.69–12.71	<0.001	2.93	1.20–7.14	0.018
∆KOOS Symptom Score	2.96	1.79–4.88	<0.001	2.61	1.53–4.43	<0.001	3.30	1.48–7.33	0.003
∆KOOS Daily Activities Score	5.94	3.22–10.96	<0.001	8.53	3.72–19.59	<0.001	7.07	1.79–27.93	0.005
∆KOOS Sport Score	3.37	2.23–5.12	<0.001	2.63	1.76–3.91	<0.001	2.30	1.35–3.92	0.002
∆KOOS QOL Score	6.39	3.35–12.19	<0.001	4.88	2.53–9.42	<0.001	2.83	1.24–6.45	0.013

## Data Availability

Data supporting the findings of this study are available from the corresponding author upon reasonable request.

## Supplementary Materials

The following supporting information can be downloaded at: https://www.mdpi.com/article/10.3390/jcm14134549/s1, Table S1: Differences in baseline characteristics between the patients who completed the 3-year follow-up visit and those who dropped out for each score.

## Abbreviations

The following abbreviations are used in this manuscript:

OA	osteoarthritis
MFAT	micro-fragmented adipose tissue
KOA	knee osteoarthritis
VAS	Visual Analog Scale
OKS	Oxford Knee Score
WOMAC	Western Ontario and McMaster Universities Osteoarthritis Index
KOOS	Knee Injury and Osteoarthritis Outcome Score
HOA	hip osteoarthritis
MSCs	mesenchymal stem cells
GMC	General Medical Council
NIHR	National Institute for Health and Care Research
SD	standard deviation
ROC	receiver operating characteristic
TKR	total knee replacement
