# Supplementary material for: Predicting Long-Term Benefits of Micro-Fragmented Adipose Tissue Therapy in Knee Osteoarthritis: Three-Year Follow-Up on Pain Relief and Mobility
Source: J Clin Med. 2025 Jun 26;14(13):4549. doi: 10.3390/jcm14134549 (PMC12249884; doi:10.3390/jcm14134549)
Supplement: Supplementary file 1 [file jcm-14-04549-s001.zip › jcm-3635765-supplementary.pdf]

Supplementary Table S1. Differences in baseline characteristics between the patients who completed the 3-year follow-up visit and those who dropped out for each score.

**VAS Pain Score**		**3 Years Complete Group (n=106)**	**3 Years Dropout Group (n=229)**	**P Value**
Age, mean±SD		63.19±11.45	65.36±11.34	0.206
Male, no. (%)		46 (43.4%)	140 (61.1%)	0.002
BMI, mean±SD		27.12±4.07	27.77±4.88	0.525
Knee	Right, no. (%)	55 (51.9%)	119 (52.0%)	0.989
	Left, no. (%)	51 (48.1%)	110 (48.0%)	
Kellgren-Lawrence Classification	Not available, no. (%)	3 (2.8%)	3 (1.3%)	0.861
	Grade I, no. (%)	6 (5.8%)	10 (4.4%)	
	Grade II, no. (%)	19 (18.4%)	49 (21.6%)	
	Grade III, no. (%)	25 (24.3%)	58 (25.6%)	
	Grade IV, no. (%)	53 (51.5%)	109 (48.0%)	
Pre-Operative Score, mean±SD		45.30±27.17	42.65±25.87	0.485
**OKS Score**		**3 Years Complete Group (n=59)**	**3 Years Dropout Group (n=276)**	**P Value**
Age, mean±SD		63.80±11.49	64.86±11.39	0.578
Male, no. (%)		31 (52.5%)	155 (56.2%)	0.612
BMI, mean±SD		26.96±4.21	27.71±4.73	0.470
Knee	Right, no. (%)	34 (57.6%)	140 (50.7%)	0.335
	Left, no. (%)	25 (42.4%)	136 (49.3%)	
Kellgren-Lawrence Classification	Not available, no. (%)	-	6 (2.1%)	0.279
	Grade I, no. (%)	5 (8.5%)	11 (4.1%)	
	Grade II, no. (%)	12 (20.3%)	56 (20.7%)	
	Grade III, no. (%)	19 (32.2%)	64 (23.6%)	
	Grade IV, no. (%)	23 (39.0%)	139 (51.3%)	
Pre-Operative Score, mean±SD		31.93±9.49	30.04±8.76	0.103
**WOMAC Score**		**3 Years Complete Group (n=54)**	**3 Years Dropout Group (n=281)**	**P Value**
Age, mean±SD		63.98±10.98	64.80±11.49	0.702
Male, no. (%)		27 (50.0%)	159 (56.6%)	0.373
BMI, mean±SD		26.73±4.19	27.74±4.72	0.261
Knee	Right, no. (%)	29 (53.7%)	145 (51.6%)	0.777
	Left, no. (%)	25 (46.3%)	136 (48.4%)	
Kellgren-Lawrence Classification	Not available, no. (%)	-	6 (2.1%)	0.214
	Grade I, no. (%)	5 (9.3%)	11 (4.0%)	
	Grade II, no. (%)	12 (22.2%)	56 (20.3%)	
	Grade III, no. (%)	17 (31.5%)	66 (23.9%)	
	Grade IV, no. (%)	20 (37.0%)	142 (51.4%)	
Pre-Operative Score, mean±SD		26.77±17.83	32.27±17.37	0.037
**KOOS Score**		**3 Years Complete Group (n=54)**	**3 Years Dropout Group (n=281)**	**P Value**
Age, mean±SD		63.98±10.98	64.80±11.49	0.702
Male, no. (%)		27 (50.0%)	159 (56.6%)	0.373
BMI, mean±SD		26.73±4.19	27.74±4.72	0.261
Knee	Right, no. (%)	29 (53.7%)	145 (51.6%)	0.777
	Left, no. (%)	25 (46.3%)	136 (48.4%)	
	Not available, no. (%)	-	6 (2.1%)	0.214
	Grade I, no. (%)	5 (9.3%)	11 (4.0%)	

Kellgren-Lawrence Classification	Grade II, no. (%)	12 (22.2%)	56 (20.3%)	
	Grade III, no. (%)	17 (31.5%)	66 (23.9%)	
	Grade IV, no. (%)	20 (37.0%)	142 (51.4%)	
KOOS Pain Score, mean±SD		63.25±19.38	60.15±18.75	0.301
KOOS Symptom Score, mean±SD		67.33±19.82	61.61±18.76	0.051
KOOS Daily Activities Score, mean±SD		70.96±20.48	65.50±18.93	0.045
KOOS Sport Score, mean±SD		41.73±26.31	33.60±24.73	0.039
KOOS QOL Score, mean±SD		40.31±21.72	36.35±19.73	0.275
